# Self-Awareness of Sleep Apnea Symptoms Among Middle-Aged and Elderly People in Taiwan

**DOI:** 10.3389/fpsyt.2022.936097

**Published:** 2022-07-22

**Authors:** Meng-Lun Hsueh, Din Jong

**Affiliations:** ^1^Graduate Institute of Intelligent Robotics, Hwa Hsia University of Technology, New Taipei City, Taiwan; ^2^Digital Design and Information Management, Chung Hwa University of Medical Technology, Tainan City, Taiwan

**Keywords:** daytime sleepiness, sleep quality, attention, sleep apnea symptoms, self-awareness

## Abstract

In recent years, the proportion of middle-aged and elderly people in Taiwan has gradually increased and has already surpassed that of advanced countries such as Europe, the United States and Japan, therefore, the health of middle-aged and elderly people is a topic that needs attention. This is because physical deterioration or illness can lead to a decline in quality of life and create a medical burden on the individual and society. This study investigated the common symptoms of sleep apnea in middle-aged and elderly people (over 40 years old), and developed a self-test subjective perception model, using “daytime sleepiness” and “sleep quality” as influencing factors, and “attention” as mediating variables to verify the effect with sleep apnea symptoms. An online electronic questionnaire was conducted and distributed through social media and groups of friends in Taiwan. A total of 541 valid questionnaires were collected and analyzed in three stages: Descriptive Analysis, Measurement Model Validation, and Structural Equation Model. The research processes of the study showed that the sample fitted the normal distribution and the measurement model conformed with convergent reliability and discriminant validity. The research results were found that “sleep quality” had a significant negative effect on sleep apnea symptoms. “Daytime sleepiness” had a positive effect on sleep apnea symptoms. “Daytime sleepiness” had a negative effect on sleep apnea symptoms through the “attention” mediator. Finally, through the questionnaire, we hope to make the middle-aged people aware of themselves, so that they can seek early medical treatment if there are signs and symptoms of sleep apnea symptoms.

## Introduction

Due to the progress of society and the development of technology, the pace of life is accelerating, work and life are increasingly stressful, and Internet entertainment has entered everyone's life, which in turn affects the sleep situation of modern people. Poor sleep affects the body's health, causing molecular, immune, and neurological changes that can lead to cardiovascular and metabolic diseases through changes in biological processes, resulting in shorter life expectancy. Sleep deprivation also affects cognitive and athletic performance, increasing the risk of fatalities such as road safety and workplace injuries (1). Although the physio pathological significance of sleep is not well understood, many studies have confirmed that sleep has a profound effect on physiological and cognitive functions (2–4).

Sleep apnea is a poorly diagnosed and prevalent disorder, a chronic disorder of breathing during sleep that leads to daytime sleepiness, poor sleep, and quality of life. To date, its health effects have not been well addressed in the available literature (5). It is usually caused by repetitive upper respiratory tract obstruction and the main symptoms are snoring, daytime sleepiness, and decreased cognitive function. It causes cardiovascular disease, pulmonary disease, and increased mortality, which increases with age (6). Many people think that sleep apnea does not require treatment, but in fact, if the disease is left untreated, it may affect life at best and harm health at worst. If the disease is not evaluated and treated immediately, it can cause complications, poor work efficiency, and an additional financial burden on the individual's society. One of the main reasons for this is that it is very difficult to notice that people are suffering from sleep apnea If a subjective questionnaire for sleep apnea can be developed in the form of a questionnaire to serve as a precursor to the physician's diagnosis, it can help the physician and the patient to confirm the patient's diagnosis of sleep apnea as early as possible. In Taiwan, nearly one million adults with sleep apnea have not yet been effectively detected, mainly because of the current testing method and standard diagnostic method–polysomnography (PSG), which requires the subject to sleep overnight in a sleep center and includes tests such as oronasal flow by nasal cannula, thoracoabdominal movements, electrocardiography, submental and pretibial electromyography, electrooculography, electroencephalography and pulse oximetry (7).

However, this approach can be costly because it requires continuous sleep monitoring by medical staff and limited capacity due to bed space and queuing, which can affect willingness to be tested. Alternative diagnostic methods include “the Home Sleep Test (HST),” in which the subjects take the device home and set up the device themselves when they go to bed. During the HSTs test, the patient should be asleep. Effort (inductive plethysmography), airflow (thermal and nasal pressure), and oxygen saturation were adopted for the testing. However, EEG was not adopted. Sleep cannot be recorded (8).

The advantage is that it can be performed in the patient's own bed, which is closer to the actual sleep situation; the disadvantage is that it is not possible to diagnose accurately if the patient has comorbidities and is therefore limited. As a result, various questionnaire methods have been developed for screening and have been shown to be suitable for predicting symptoms of sleep apnea, including the Berlin Questionnaire, the Epworth Sleepiness Scale, and the STOP-Bang questionnaire. However, current screening methods have high sensitivity but low specificity, and it is difficult to obtain accurate results that can be trusted, so new questionnaire screening methods need to be developed (9). In particular, there is a need to develop questionnaires that are scaled for subjective patient perception judgments, which allow early detection of symptoms and further early treatment.

This study will develop a research model for the subjective detection of sleep apnea factors by patients. Based on the established scales, we developed a suitable questionnaire and distributed it to collect information for the study. To investigate the subjective important factors of apnea in middle-aged adults in Taiwan, including daytime sleepiness, sleep quality, attention, and self-awareness of apnea characteristics, by statistically verifying the relationship between the factors, in order to alert middle-aged adults for early detection of apnea and early treatment. Through proper control, the patients can improve their sleep and life quality.

## Literature Review and Hypotheses Development

The section is divided into five parts: daytime sleepiness, attention, sleep quality, sleep apnea symptoms, and correlation between the variables.

### Daytime Sleepiness

The term sleepiness did not appear in the index of medical textbooks in the past, so it is still a concept that is not widely used or clearly understood. Different people have different views of what sleepiness is, leading some to debate whether sleepiness can be defined by one or more measurements or tests.

Some people consider the subjective definition of sleepiness to be a state of feeling tired or fatigued on a regular basis and therefore feeling the need to sleep, as measured by the Stanford Sleepiness Scale (SSS). In addition, sleepiness, as measured by the visual analog scale (VAS), refers to a state of low alertness and fatigue represents a possible need for sleep, but neither of these provides a useful measure of sleepiness. Furthermore, some define sleepiness as a physiological drive caused by sleep deprivation, a state of physiological demand that leads to an increased tendency to fall asleep, the ease with which sleep is disrupted, or the duration and state of sleep, which can be inferred from the presence of sleepiness (10). Finally, the Epworth Sleepiness Scale (ESS) is a popular method used in most countries to assess excessive daytime sleepiness. Studies have demonstrated the reliability and validity of the ESS scale as an acceptable, reliable, and valid measure of daytime sleepiness (11), which is subjectively defined by the concept of daytime sleepiness as a self-reported tendency to fall asleep unintentionally or increased difficulty in staying awake (12). When the ESS exceeds 11 points, the subject can be said to have signs of daytime sleepiness (13).

There is semantic confusion between the concepts of “fatigue” and “sleepiness,” and the Epworth Sleepiness Scale (ESS) assesses that fatigue accounts for a large proportion of daytime sleepiness in individuals. There was a moderate association between ESS scores and various fatigue scales. Second, daytime sleepiness is often overlooked in the treatment of patients with multiple sclerosis and may also be related to sleep disorders. Sleepiness and potential sleep disorders may lead to fatigue and perpetuate fatigue, sleepiness should be part of the diagnosis (14). In addition, studies have demonstrated that daytime sleepiness and impaired sleep quality are associated with reduced self-management in adults with diabetes (12). Furthermore, excessive daytime sleepiness is a common disorder throughout the world and can have a negative impact on health and quality of life.

### Attentions

Attention is the ability to select and focus on meaningful stimuli. Most definitions of attention relate to the selective processing of information. The biased-competition theory defines signal competition as the information gained by the people inside their brains. The winning signal guides people's behavior and reaction. Response accuracy and reaction time are associated with the attention dimension (15). Attention refers to a variety of phenomena, including focus, selectivity, automaticity, conscious monitoring, and capacity limitations. Second, current research on attention distinguishes between selective attention to perceived information and higher-order focused executive functions that are involved in planning and organizing complex actions (16). Furthermore, neuropsychological research clearly shows that attentional capacity is based on a variety of processes. These processes control the flow of information to cognitive systems that have recently been defined in anatomical and functional terminology. Therefore, maintaining adequate attention allows the application of different cognitive processes and requires different tests dedicated to these different processes to adequately assess attention (17).

Attention deficit and hyperactivity disorder (ADHD) is the most common neurobehavioral disorder in childhood. In 30% to 70% of cases, ADHD has been shown to persist into adulthood (17). It is well known that persistent ADHD is a serious risk factor for other diseases in adulthood, and adults with ADHD often report sleep disorders. Despite this, only a few studies have investigated the subjective and objective sleep quality of adults with ADHD (18). Severe obstructive sleep apnea (OSA) directly affects an individual's quality of life, mood, and sustained attention, and it is known from the literature that patients with mild OSA exhibit sustained attention deficits compared to normal subjects (19).

### Sleep Quality

Sleep can be defined as a regular, repetitive, and easily reversible state of the organism, characterized by relative stillness and a significant increase in the threshold of response to external stimuli compared to the waking state. Quality is defined as the degree of excellence of something. That is, sleep quality is the degree of excellence in sleep. Many researchers have measured it according to this definition. Based on a literature review of previous questionnaires and studies, the sleep quality domains were identified as sleep onset, sleep maintenance, sleep depth, dreams, getting up after sleep, post-sleep status, impact on daily life, amount of sleep, and satisfaction with sleep (20). Good sleep quality is conceptualized as falling asleep quickly, getting enough sleep without interruptions, waking up feeling refreshed, and staying alert during the day. The Pittsburgh Sleep Quality Index (PSQI) is a 19-item validated questionnaire that can be used to differentiate between “good” and “poor” sleepers. PSQI scores were obtained from the components of subjective sleep quality, sleep latency, sleep duration, habitual sleep efficiency, sleep disturbance, sleeping pill use, and daytime dysfunction. Answers are rated on a scale of 0 to 3, where 3 represents the negative end of the scale on the Likert scale. Higher scores indicate poor sleep quality. PSQI cumulative total score >5 indicates poor sleep quality (12).

Subjective sleep quality is an important determinant of health-related quality of life. Many scholars have conducted different studies, for example, in Lee, W., self-assessed sleep quality was found to be closely related to the quality of life in a healthy Austrian population. However, there are relatively few studies on the relationship between sleep quality and quality of life in the obstructive sleep apnea population (13). Second, sleep quality affects health and overall quality of life. Since the factors that affect sleep quality and their relative importance vary from person to person, self-reporting methods are essential. Although various questionnaires have been used to assess sleep quality, few comprehensive assessment scales have been developed (20).

Poor sleep quality has been shown to reduce cognitive performance, impair psychosocial functioning, and alter perceptions of stress. However, little is known about how sleep quality affects emotion processing. Studies have indicated that the extent to which sleep quality can influence emotional symptoms and the interaction between stress and performance on emotional memory tests and sustained attention tasks can be measured by the Pittsburgh Sleep Quality Index (21).

### Sleep Apnea Symptoms

Sleep apnea is a very common chronic sleep disorder. Recurring airway obstruction leads to irregular decreases in oxygen saturation and sleeps arousals, resulting in fragmented sleep (13, 22, 23). Sleep-related seizure disorders that do not reply to conventional therapy, sleep-related breathing disorders, periodic limb movement during sleep, narcolepsy, parasomnias, depression with insomnia, circadian rhythm disorders, other respiratory disorders, and sleep-related symptoms related to neuromuscular disorders are various types of sleep disorders (15). Obstructive sleep apnea (OSA) is well known to be characterized by polysomnography (PSG), including recurrent electroencephalogram (EEG) arousals after disruption of airflow during sleep, and PSG is the standard by which OSA is diagnosed (23). OSA has a variety of symptoms, including fatigue and excessive daytime sleepiness (EDS) (24).

Previous studies have shown that many questionnaires are suitable for predicting symptoms of sleep apnea syndrome. The measure of online screening questionnaire was used to conduct the study. The measure showed sufficient construct validity, internal validity, and criterion validity to suggest that it is a useful and valid screening method for sleep apnea risk in adults (25).

### Hypotheses Developments

Daytime sleepiness can be the main symptom described by patients with sleep apnea (25). The Epworth Sleepiness Scale (ESS) was used to measure daytime sleepiness. Its scores significantly differentiated between patients with primary snoring and patients with obstructive sleep apnea. ESS scores increased with the severity of sleep apnea. Multiple regression analysis also showed that ESS scores were more closely related to the frequency of respiratory pauses than to the degree of hypoxemia in OSAS (10). Therefore, daytime sleepiness should have a positive effect on sleep apnea symptoms, and for middle-aged and elderly people, the higher the level of daytime sleepiness, the higher the level of sleep apnea symptoms. Therefore, daytime sleepiness in the middle-aged group would have a positive and significant effect on sleep apnea symptoms. This study proposed the following hypothesis:

**H1: Daytime sleepiness has a positive and significant effect on obstructive sleep apnea symptoms**.

Some studies have shown that daytime sleepiness is associated with attention, memory, reaction time, problem-solving ability, and cognitive ability. That reduced attention increases the risk of accidents. Attention was significantly correlated with age and daytime sleepiness (26). Therefore, daytime sleepiness affects attention. For middle-aged and elderly people, the higher the level of daytime sleepiness, the lower the level of attention. This study hypothesizes that daytime sleepiness has a negative significant effect on attention in middle-aged and elderly people. The following hypothesis is proposed:

**H2: Daytime sleepiness has a negative significant effect on attention**.

In addition, the lower the attention span may be due to sleep apnea symptoms, when patients notice a decline in their attention span, it is likely that they are suffering from sleep apnea. Therefore, this study hypothesizes that the attention of middle-aged and elderly people has a negative significant effect on the symptoms of sleep apnea. The following hypothesis is proposed:

**H3: Attention has a negative significant effect on obstructive sleep apnea symptoms**.

Studies have shown that professional drivers are prone to daytime sleepiness and poor sleep quality (27). Risk factors for excessive sleepiness are obesity, anxiety, age, and sleep deprivation (28). This often leads to obstructive sleep apnea and periodic limb movement disorder in the clinical.

For middle-aged and elderly people, the higher the degree of daytime sleepiness, the lower the sleep quality should be. Therefore, this study concludes that daytime sleepiness in middle-aged and elderly people has a negative and significant effect on sleep quality. The following research hypothesis is proposed:

**H4: Daytime sleepiness has a negative and significant effect on sleep quality**.

In terms of sleep quality and attention, it has been suggested that 30% of children and 60–80% of adults with attention deficit and hyperactivity disorder (ADHD) have symptoms of sleep disturbance, that sleep causes or exacerbates symptoms of ADHD, and that good sleep quality improves attention (28). Therefore, this study hypothesizes that sleep quality has a positive effect on attention in middle-aged and elderly people, and the hypothesis is as follows:

**H5: Sleep quality has a positive and significant effect on attention**.

Patients with sleep apnea suffer from sleep fragmentation and sleep-wakefulness, so the quality of sleep is generally low, and sleep quality has fewer sleep apnea symptoms (29). For middle-aged and elderly people, the better the sleep quality, the lower the level of sleep apnea symptoms. Therefore, this study hypothesizes that the sleep quality of middle-aged and elderly people has a negative and significant effect on the symptoms of sleep apnea:

**H6: Sleep quality has a negative and significant effect on the sleep apnea symptoms**.

In summary, daytime sleepiness had a negative effect on attention, and attention also had a negative effect on sleep apnea symptoms. Therefore, this study hypothesizes that daytime sleepiness in the middle-aged and elderly populations will affect the symptoms of obstructive sleep apnea through attention:

**H7: The attention will have a mediation effect between daytime sleepiness and apnea symptoms**.

Also, sleep quality had a positive effect on attention and attention had a negative effect on apnea symptoms. Therefore, this study hypothesizes that sleep quality in the middle-aged and elderly groups will have an effect on apnea symptoms through attention, and proposes the following hypothesis:

**H8: The attention will have a mediation effect between sleep quality and apnea symptoms**.

**Figure 1:**
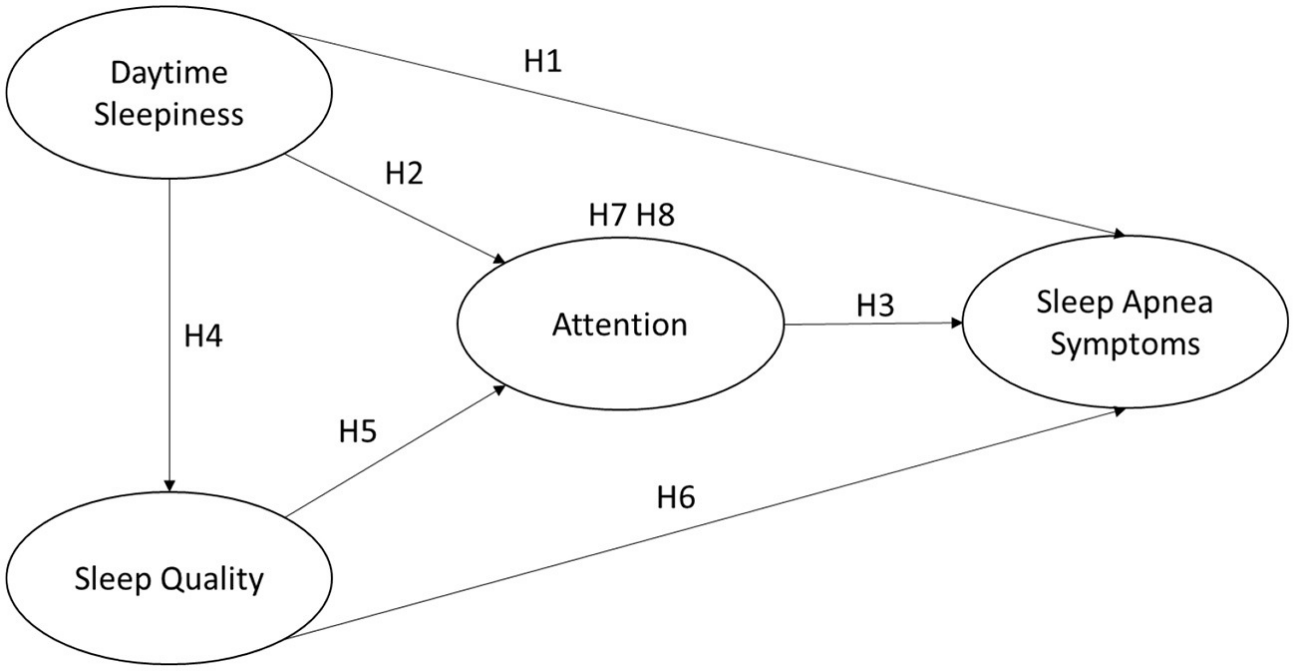
Research framework.

This study was conducted to investigate the self-awareness of sleep apnea symptoms in middle-aged and elderly people, and to study the influencing factors. In this study, “daytime sleepiness,” “attention,” and “sleep quality” were used as independent variables, with “attention” also playing the role of a mediating variable, and “sleep apnea symptoms” as a dependent variable. The research framework of this study was developed from the literature as shown in Figure 1.

**Figure 2:**
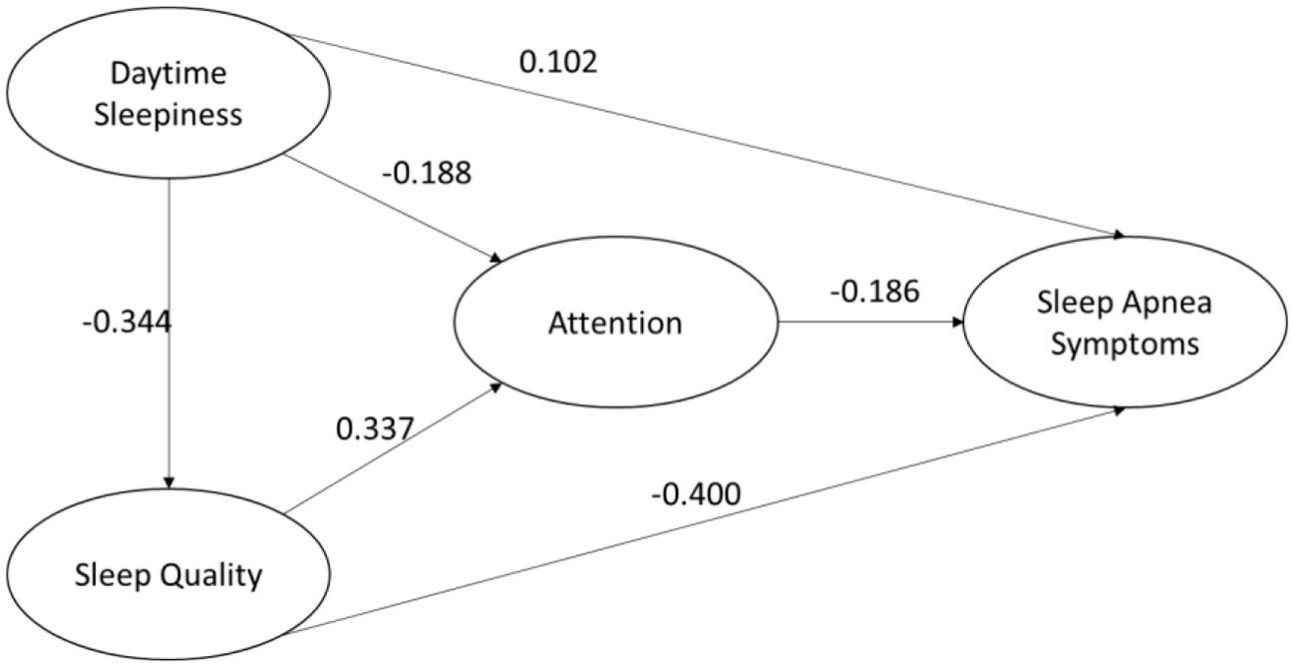
Statistical model diagram.

## Research Design

### Research Subjects and Data Collection

In this study, the research purpose is to investigate the influence of self-awareness on sleep apnea symptoms among middle-aged and elderly people in Taiwan. The study population was set at 40 years old and above. In this study, an online survey was conducted by means of an online e-questionnaire, which was distributed through social media and groups of friends in Taiwan. 592 questionnaires were collected during the period from 2022/01/10 to 2022/02/10, and after removing invalid questionnaires, the total number of valid questionnaires was 541. According to the sample size requirement formula of The Survey System website (https://www.surveysystem.com/sscalc.htm), with a statistical confidence level of 95% and a confidence interval of 5%, the sample size of the total population in Taiwan is 384, which shows that the sample size of this study is sufficient.

### Description of Research Variables

The study variables include basic personal background, daytime sleepiness, attention, sleep quality, and apnea symptoms. This study proposes a multivariate linear regression formula, with sleep apnea as the dependent variable in this study, represented by Y. The independent variables include daytime sleepiness, attention and sleep quality, represented by X_1_, X_2_ and X_3_. β_0_ is the intercept of the regression model and ε is the error term. β_1_, β_2_, and β_3_ represent their independent variable regression coefficients respectively. The proposed formula is as follows:


$$\begin{array}{l} Y=\beta_{0}+\beta_{1}X_{1}+\beta_{2}X_{2+}\beta_{3}X_{3+}\varepsilon . \end{array}$$

#### Basic Background

In this study, the possible influences of self-perception of sleep apnea symptoms and the importance of their influence in middle-aged and elderly people in Taiwan were investigated. Therefore, basic personal information was measured in the first part of the survey, including gender, age, marital status, and education level.

#### Daytime Sleepiness

This study used the study of Johns in 1991 (30) for the construct of daytime sleepiness, which has been developed over the years and is a very mature topic. It provides a simple standardized method to measure sleepiness, as opposed to objective methods of sleepiness examination which can be complex and costly. This is a short and easy-to-administer questionnaire that can subjectively measure drowsiness in the subject's daily life, and the questions are measured on a 4-point scale ranging from no dozing (0), rarely dozing (1), occasionally dozing (2), and often dozing (3), etc. There are 8 questions in the questionnaire, and the normal score range of the questionnaire is 0 to 24, and a score >10 will require further medical evaluation (31). This questionnaire has a high consistency among the 8 items (10) as shown in Table 1.

**Table 1:** Questionnaire items.

	**Source**
**Daytime sleepiness**	
1. Sitting and reading	Johns (30)
2. When watching TV	
3. Sitting, inactive, in a public place (e.g., in a meeting, theater, or dinner event).	
4. As a passenger in a car for an hour or more without stopping for a break	
5. Lying down to rest when circumstances permit	
6. Sitting and talking to someone	
7. Sitting quietly after a meal without alcohol	
8. In a car, while stopped for a few minutes in traffic or at a light	
**Attention**	
1. I could be experiencing some emotion and not be conscious of it until sometime late	Brown and Ryan (32)
2. I break or spill things because of carelessness, not paying attention, or thinking of something else.	
3. I find it difficult to stay focused on what's happening in the present.	
4. I tend to walk quickly to get where I'm going without paying attention to what I experience along the way.	
5. I tend not to notice feelings of physical tension or discomfort until they really grab my attention	
6. I forget a person's name almost as soon as I've been told it for the first time.	
7. It seems I am “running on automatic,” without much awareness of what I'm doing.	
8. I rush through activities without being really attentive to them.	
9. I do jobs or tasks automatically, without being aware of what I'm doing.	
10. I find myself listening to someone with one ear, doing something else at the same time.	
11. drive places on “automatic pilot” and then wonder why I went there.	
12. I find myself preoccupied with the future or the past.	
13. I find myself doing things without paying attention.	
14. I snack without being aware that I'm eating.	
**Sleep quality**	
1.During the past month, what time have you usually gone to bed at night?	Shahid et al. (33)
2.During the past month, how long (in minutes) has it usually takes you to fall asleep each night?	
3.During the past month, what time have you usually gotten up in the morning?	
4.During the past month, how many hours of actually sleep did you get at night?	
5.During the past month, how often have your had trouble sleeping by the following? (Unable to sleep within 30 min, wake up in the middle of the night or early morning, have to get up to use the bathroom, have trouble to breathe comfortably, cough or snore loudly, feel too cold, feel too hot, have bad dreams, pain and other conditions)	
6.During the past month, how often have you taken medicine (doctor's prescriptions or prescription drugs) to help you sleep?	
7.During the past month, how often have you had trouble staying awake while driving, eating meals, or engaging in social activity?	
8.During the past month, how much of a problem has it been for you to keep up enough enthusiasm to get things done?	
9. During the past month, how would you rate your sleep quality overall?	
**Sleep apnea symptoms**	
1. I often snore when I sleep and it is very loud.	Chung et al. (34)
2. I often feel fatigued.	
3. I would choke or gasp when I slept.	
4. I have high blood pressure and am being treated for it.	

#### Attention

The present study on the constructs of attention refers to Brown and Ryan (32), Carlson (35), a 15-item scale that focuses on the core characteristics of attention. Attention is a receptive state of mind in which it is possible to perceive what is happening and to observe what is going on–the questionnaire questions in this study invited expert scholars to examine the questions and give their opinions. “I get so focused on the goal I want to achieve that I lose touch with what I am doing right now to get there. “and” It seems I am “running on automatic,” without much awareness of what I'm doing.” The previous item was deleted because it was too close. The questions were scored on a six-point scale ranging from almost always (1), very frequently (2), somewhat frequently (3), somewhat infrequently (4), very infrequently (5), and almost never (6), with 14 questions, as shown in Table 1.

#### Sleep Quality

The study of Shahid et al. (33) for the dimensions of sleep quality was referenced. Among 19 self-reported questions in the study, seven of them were adopted, including subjective sleep quality, sleep latency, sleep duration, habitual sleep efficiency, sleep disturbance, use of sleeping pills and daytime dysfunction, which were revised according to the content and discussed with academic experts. This study developed two more questions for the measurement. A total of nine questions were designed, as shown in Table 1. There are four options: more than three times per week, 1–2 times per week, <1 time per week, and never.

#### Sleep Apnea Symptoms

In this study, the constructed questionnaire for sleep apnea symptoms was modified with reference to the questionnaire in the study of Chung et al. (34), Kline (36). The question measure was scored on a seven-point Likert scale ranging from strongly disagree (1), disagree (2), somewhat disagree (3), average (4), somewhat agree (5), agree (6), and strongly agree (7). The higher the score, the higher the level of agreement with the study variable. After the design of the questionnaire questions, experts were invited to review the questions and give their opinions. The design of this structure provides 4 questions, as shown in Table 1.

### Data Analysis

The results of this study are divided into three parts: descriptive statistical analysis, measurement model validation, and structural equation modeling analysis. Structural Equation Model (SEM) was used to obtain relevant conclusions. Most of the questionnaires used continuous scales, which are statistically consistent with the normal distribution and yielded more accurate results. The descriptive statistical analysis in SPSS consists of two parts: one is the calculation of the frequency distribution of the statistics in order to have a basic understanding of the sample set; the other is the mean and standard deviation of each constructed item. Then, according to Anderson and Gerbing (37), the study validated the measurement model by confirmatory reliability analysis, convergent validity, and discriminant validity. Subsequently, the structural equation model was analyzed on the basis of the research model, including path analysis and mediating effects analysis through the statistical software AMOS (SPSS Inc., Chicago, IL, USA).

## Research Results

### Normally Distributed Analysis

The average values are all between 1.56 and 4.26. The standard deviations of all questions are between 0.705 and 1.918, showing the consistency of each question that the participants responded.

The skewness ranged from −1.201 to 1.582, and the kurtosis valued from-1.207to 1.636, which is consistent with the suggestions that the absolute value of skewness is less than 2 and the absolute value of kurtosis is <7 (36). Thus, the data is normally distributed as shown in Table 2.

**Table 2:** Normally distributed analysis.

**Item**	**Mean**	**Standard deviations**	**Skewness**	**Kurtosis**
DS 1	2.47	0.792	−0.253	−0.480
DS 2	2.43	0.931	−0.085	−0.907
DS 3	2.19	0.931	0.298	−0.824
DS 4	2.71	0.975	−0.396	−0.803
DS 5	2.96	0.981	−0.742	−0.408
DS 6	1.61	0.882	1.406	1.111
DS 7	2.23	0.963	0.231	−0.965
DS 8	1.56	0.871	1.582	1.636
ATT1	3.96	1.324	−0.100	−0.944
ATT2	3.89	1.344	−0.073	−0.891
ATT3	4.05	1.330	−0.222	−0.936
ATT4	3.52	1.402	0.299	−0.793
ATT5	3.61	1.452	0.132	−0.983
ATT6	3.46	1.446	0.355	−0.840
ATT7	3.98	1.268	−0.045	−0.777
ATT8	3.92	1.272	−0.142	−0.811
ATT9	3.70	1.337	0.131	−0.772
ATT10	3.60	1.435	0.203	−0.987
ATT11	4.26	1.408	−0.365	−0.932
ATT12	3.64	1.385	0.225	−0.959
ATT13	3.77	1.329	0.104	−0.927
ATT14	4.17	1.382	−0.273	−0.963
SQ1	2.67	0.944	−0.128	−0.912
SQ2	2.55	0.985	0.045	−1.036
SQ3	2.61	0.999	−0.002	−1.098
SQ4	3.21	0.933	−0.817	−0.512
SQ5	2.84	1.002	−0.324	−1.038
SQ6	2.84	0.948	−0.259	−0.967
SQ7	3.04	0.861	−0.469	−0.643
SQ8	2.98	0.839	−0.419	−0.528
SQ9	3.16	0.898	−0.707	−0.541
SQ10	3.38	0.945	−1.201	0.030
SQ11	3.23	0.873	−0.760	−0.554
SQ12	2.71	0.827	0.047	−0.745
SQ13	2.82	0.705	−0.184	−0.146
SA1	3.39	1.918	0.217	−1.207
SA2	4.17	1.825	−0.407	−0.886
SA3	2.91	1.613	0.534	−0.637
SA4	2.37	1.772	1.147	0.106

*DS, Daytime Sleepiness; ATT, Attention; SQ, Sleep Quality; SA, Sleep apnea*.

### Descriptive Statistics

Regarding the distribution of the sample in this study, in terms of gender, 338 females accounted for 62.5% of the total; 436 aged 40–50 accounted for 80.6%; 368 married accounted for 68.0%; and 370 educational attainment accounted for 68.4% of the total (Table 3).

**Table 3:** Frequency distribution table.

**Variable**	**Value label**	**Frequency**	**Percent**
Gender	Male	203	37.5
	Female	338	62.5
Age	40–50 years old	436	80.6
	51–60 years old	75	13.9
	61–70 years old	29	5.4
	Over 71 years old	1	0.2
Marriage	Married	368	68.0
	Unmarried	173	32.0
Education	Elementary school	7	1.3
	Junior High School	18	3.3
	Senior High School	99	18.3
	College and University	370	68.4
	Master's degree or above	47	8.7

### Convergent Validity and Discriminant Validity

This study follows the two-step approach to estimate the measurement and structural model proposed by Anderson and Gerbing (37) for structural equation modeling (SEM). The first step uses confirmatory factor analysis (CFA) to check the construct reliability and validity of the measurement model, and the second step checks the path effects of the structural model and its significance. In this study, a structured two-step approach with maximum likelihood estimation (MLE) was used to assess the factor loadings, reliability of the construct, convergent validity, and discriminant validity of the measurement models.

Table 4 presents a summary of unstandardized factor loadings, standard errors, significance tests, standardized factor loadings, squared multiple correlations, composite reliability, and average variance extracted (AVE).

**Table 4:** Results for the measurement model.

**Construct**	**Item**	**Significance of estimated parameters**				**Item Reliability**		**Construct Reliability**	**Convergence validity**
		**Unstd**.	**S.E**.	**Unstd. / S.E**.	***p*-value**	**Std**.	**SMC**	**CR**	**AVE**
Daytime Sleepiness	DS1	1.000				0.892	0.796	0.918	0.586
	DS2	1.097	0.042	26.252	0.000	0.832	0.692		
	DS3	1.035	0.045	23.208	0.000	0.785	0.616		
	DS4	1.015	0.048	21.180	0.000	0.736	0.542		
	DS5	0.985	0.049	20.082	0.000	0.709	0.503		
	DS6	0.858	0.046	18.479	0.000	0.687	0.472		
	DS7	1.098	0.045	24.419	0.000	0.805	0.648		
	DS8	0.802	0.047	17.077	0.000	0.650	0.423		
ATT	ATT1	1.000				0.837	0.701	0.965	0.666
	ATT2	1.024	0.041	25.159	0.000	0.844	0.712		
	ATT3	1.043	0.040	26.406	0.000	0.869	0.755		
	ATT4	0.950	0.046	20.819	0.000	0.752	0.566		
	ATT5	1.012	0.047	21.759	0.000	0.773	0.598		
	ATT6	0.982	0.047	20.907	0.000	0.753	0.567		
	ATT7	1.034	0.037	28.212	0.000	0.904	0.817		
	ATT8	0.994	0.038	26.194	0.000	0.866	0.750		
	ATT9	0.995	0.041	24.067	0.000	0.825	0.681		
	ATT10	0.853	0.049	17.357	0.000	0.659	0.434		
	ATT11	1.040	0.044	23.855	0.000	0.819	0.671		
	ATT12	0.987	0.044	22.427	0.000	0.790	0.624		
	ATT13	1.049	0.039	26.639	0.000	0.875	0.766		
	ATT14	1.031	0.043	24.241	0.000	0.827	0.684		
SQ	SQ1	1.000				0.746	0.557	0.945	0.572
	SQ2	1.000				0.715	0.511		
	SQ3	0.990	0.059	16.675	0.000	0.698	0.487		
	SQ4	1.015	0.056	18.138	0.000	0.766	0.587		
	SQ5	0.987	0.060	16.408	0.000	0.694	0.482		
	SQ6	0.972	0.057	17.173	0.000	0.722	0.521		
	SQ7	0.946	0.051	18.366	0.000	0.773	0.598		
	SQ8	0.944	0.050	19.032	0.000	0.792	0.627		
	SQ9	0.999	0.053	18.716	0.000	0.784	0.615		
	SQ10	0.962	0.057	16.925	0.000	0.717	0.514		
	SQ11	0.918	0.053	17.478	0.000	0.741	0.549		
	SQ12	0.950	0.048	19.834	0.000	0.809	0.654		
	SQ13	0.855	0.041	20.914	0.000	0.854	0.729		
SA	SA1	1.000				0.700	0.490	0.836	0.564
	SA2	0.908	0.063	14.336	0.000	0.668	0.446		
	SA3	1.073	0.066	16.272	0.000	0.893	0.797		
	SA4	0.953	0.063	15.102	0.000	0.722	0.521		

*Unstd, Unstandardized factor loadings; Std, Standardized factor loadings; SMC, Square Multiple Correlations; CR, Composite Reliability; AVE, Average Variance Extracted; DS, Daytime Sleepiness; ATT, Attention; SQ, Sleep Quality; SA, Sleep apnea*.

As shown in Table 4, the Std of Item Reliability ranged from 0.65 to 0.904, which is a reasonable range. This indicates that all the questions have convergent validity. The composite reliability of all the constructs ranged from 0.836 to 0.965, exceeding the 0.7 recommended by Nunnally (38), indicating that all constructs are internally consistent. Finally, all average variance extracted (AVE) ranged from 0.564 to 0.666, exceeding the 0.5 suggested by Hair et al. (39) and Fornell and Larcker (40), indicating sufficient convergent validity for all constructs.

A comparison of the square root of the AVE with the correlation between the construct and other constructs can be used to measure the discriminant validity (40). As shown in Table 5, the bold numbers in the diagonal direction indicate the square root of the AVE. Because all numbers in the diagonal direction are larger than the non-diagonal numbers, the discriminant validity seems to be satisfactory for all constructs.

**Table 5:** Discriminant validity for the measurement model.

	**AVE**	**DS**	**ATT**	**SQ**	**SA**
DS	0.586	0.766			
ATT	0.666	−0.304	0.816		
SQ	0.572	−0.344	0.402	0.756	
SA	0.564	0.297	−0.378	−0.510	0.751

*The items on the diagonal in bold represent the square roots of the AVE; off-diagonal elements are the correlation estimates*.

*DS, Daytime Sleepiness; ATT, Attention; SQ, Sleep Quality; SA, Sleep apnea*.

### Model Fit

In this study, model fit is reported as the most widely used nine fitness metrics (41). Since the SEM sample is larger than 200, the cardinality value is too large and leads to poor fit, so the fit value needs to be corrected by Bootstrap method (42). The results of the Bollen-Stine Bootstrap modified model fit are shown in Table 6. After the Bollen-Stine Bootstrap modified model fit, the fit indicators of this study passed, indicating that the results of this study were acceptable.

**Table 6:** Model fit.

**Fit indices**	**Model fit criteria**	**Measurement model**	**Results**
Chi-square		849.246	
Degree of freedom		696	
CFI	>0.9	0.992	Pass
RMSEA	<0.08	0.020	Pass
TLI	>0.9	0.990	Pass
GFI	>0.9	0.952	Pass
NFI	>0.9	0.952	Pass
χ^2^/df	<3	1.220	Pass
AGFI	>0.8	0.946	Pass

*CFI, Comparative Fit Index; RMSEA, Root Mean Square Error of Approximation; TLI, Tucker-Lewis Index; GFI, Goodness-of-Fit Index; NFI, Normed Fit Index; AGFI, Adjusted Goodness- of-Fit Index*.

### Path Analysis

Table 7 shows the results of the path coefficients. First, daytime sleepiness (b = −0.296, *p* < 0.001) and sleep quality (b = 0.531, p <0.001) significantly affected attention. This shows that both H2 and H5 paths are valid. Then, daytime sleepiness (b = −0.343, *p* < 0.001) significantly affected sleep quality, which also showed that H4 was established. Moreover, daytime sleepiness (b = 0.194, *p* = 0.024), attention (b = −0.225, *p* < 0.001) and sleep quality (b = −0.761, *p* < 0.001) significantly affected sleep apnea symptoms. This suggests that H1, H3 and H6 is valid.

**Table 7:** Regression coefficient.

**DV**	**IV**	**Unstd**	**S.E**.	**Unstd./S.E**.	***p*-value**	**Std**.	**R^**2**^**
ATT	DS	−0.296	0.070	−4.223	0.000	−0.188	0.193
	SQ	0.531	0.073	7.247	0.000	0.337	
SQ	DS	−0.343	0.046	−7.492	0.000	−0.344	0.119
SA	DS	0.194	0.086	2.250	0.024	0.102	0.304
	ATT	−0.225	0.056	−3.990	0.000	−0.186	
	SQ	−0.761	0.100	−7.601	0.000	−0.400	

*DS, Daytime Sleepiness; ATT, Attention; SQ, Sleep Quality; SA, Sleep apnea*.

*The statistical analysis results of the research model were as shown in Figure 2*.

The above results support the validity of the research model. 19.3% of attention can be explained by daytime sleepiness and sleep quality. 11.9% of the sleep quality can be explained by daytime sleepiness construct. 30.4% of sleep apnea can be explained by daytime sleepiness, attention, and sleep quality.

### Mediation Effects

The indirect effect DS → ATT → SA, *p* < 0.05, and the deviation-corrected confidence interval (CI) did not contain 0 (CI = [0.022 0.131] for DS → ATT → SA). Therefore, the assumption of indirect effects is supported to exist. Then, the indirect effect SQ → ATT → SA, *p* < 0.05, and the deviation-corrected confidence interval (CI) did not contain 0 (CI = [−0.209–0.043] for SQ → ATT → SA). Therefore, the assumption of indirect effects is supported to exist. Moreover, the indirect effect DS → ATT → SA, p <0.05, bias-corrected confidence interval. The results were shown in Table 8.

**Table 8:** The analysis of indirect effects.

**Effect**	**Point Estimate**	**product of coefficients**			**Bootstrap 1,000 times**	
					**Bias-corrected 95%**	
		**S.E**.	**Z-Value**	***p*-value**	**Lower bound**	**Upper bound**
Specific indirect effect	
DS → ATT → SA	0.067	0.029	2.323	0.020	0.022	0.131
SQ → ATT → SA	−0.120	0.043	−2.788	0.005	−0.209	−0.043

*DS, Daytime Sleepiness; ATT, Attention; SQ, Sleep Quality; SA, Sleep apnea*.

## Discussion

### Contribution

Sleep apnea is a difficult disease to detect and diagnose because it is difficult for the patient to recognize that he or she has the disease. In particular, modern people generally have poor sleep quality, such as low concentration, daytime sleepiness, etc. These may be caused by symptoms of sleep apnea, which are often overlooked and confused. Therefore, in addition to formal clinical tests to confirm whether the disease is present, there is an urgent need for a scale that can be used by the patient to make subjective judgments, so that the patient can detect the disease early. This study investigated the factors influencing self-awareness of sleep apnea symptoms in middle-aged and elderly people in Taiwan. Daytime sleepiness, attention, and sleep quality were selected to assess the influence of apnea symptoms. The sample statistics of this study were in line with the normal distribution and the questionnaire showed convergent reliability and discriminant validity. At the same time, each hypothesis and the mediating effect in the model is supported. The research results are listed as follows.

#### The Impact of Sleep Quality

From the results of the study, daytime sleepiness, attention, and sleep quality all had significant effects on the symptoms of apnea (path coefficients of 0.102, −0.186, and −0.400, respectively). Among them, sleep quality has the most important effect on the symptoms of apnea. This is also similar to previous studies (29). This conclusion also shows that the diagnosis of sleep apnea is very difficult because most patients with sleep apnea have poor sleep quality. It is possible that modern people work and life is more stressful, the sleep quality of adults are poor. In addition, modern people due to the development of the Internet, entertainment equipment at their fingertips, connected to the Internet, playing games, browsing the web, watching short videos, etc., spent a lot of time, the rest time is not normal and other factors, will also become the result of poor sleep quality. This study found that sleep quality is the most important antecedent of sleep apnea; therefore, improving sleep quality would be a way to reduce the formation of sleep apnea.

#### The Impact of DS and SQ on Attention

DS and SQ have an effect on attention, and their path coefficients are −0.188 and 0.337, respectively. Daytime sleepiness and sleep quality can cause poor concentration. Therefore, there is a possible increase in some apnea symptoms. This study confirmed that daytime sleepiness and poor sleep quality had a significant effect on attention. The effect of sleep quality is greater than that of daytime sleepiness. The previous conclusion is similar in that attention is influenced by the quality of sleep. To improve one's attention, it is important to ensure that one has a good quality of sleep.

#### Recognizing the Importance of Attention

Attention plays an important mediating role here, and if it can be increased, the effect of apnea symptoms will be reduced. How to improve attention may be a key concern. Of all the symptoms that affect sleep apnea, attention is perhaps the most easily recognized. In particular, the patient's family members can look at small events in the patient's daily life to see if there are signs of inattention, such as forgetting easily, not paying attention to what they are doing, being easily distracted or drifting off, and so on. The patient should be reminded to seek early medical attention.

In modern times, due to the advancement of technology and the availability of audio-visual entertainment, many people sleep too late or are harsh on their sleep schedule. Whether sleep quality is the cause of apnea symptoms is debatable, but this study confirmed that sleep quality is an important attribute influencing apnea symptoms. Therefore, when people notice that their sleep quality is not good, people should pay attention to whether it is due to apnea and seek medical attention.

### Implications

Apnea symptoms are difficult to detect in their early stages. Patients may just think that they snore in their sleep like a normal aging phenomenon. They may not even be aware that they are snoring. The patient may not seek medical attention, which causes worsening apnea symptoms. Although there are sleep centers in hospitals that can provide diagnosis and treatment, patients who do not recognize that they have sleep apnea will not seek medical attention. On the other hand, the existing diagnostic method requires patients to stay overnight at the center and connect to diagnostic equipment, and patients are unable to sleep or need to wait in line for tests, which often fail to detect the real disease. The study provides a subjective and self-aware way for the general public to be aware of the signs of apnea and to detect them early for treatment. The study can also be replicated in hospitals and provided as a reference for respiratory therapists and physicians.

### Research Limitations and Future Research Development

For sleep apnea, finding the key influencing factors is not easy. Only three influences, daytime sleepiness, attention, and sleep quality, were selected for validation in this study model. There may be other influencing factors whose existence has not been verified, such as environmental factors, personal constitution, or genetic factors. Future research should include more factors to provide users with a subjective perception of detection and improve the chance of early awareness.

## Data Availability Statement

The raw data supporting the conclusions of this article will be made available by the authors, without undue reservation.

## Ethics Statement

Ethical review and approval was not required for the study on human participants in accordance with the local legislation and institutional requirements. Written informed consent for participation was not required for this study in accordance with the national legislation and the institutional requirements.

## Author Contributions

Conceptualization and investigation: M-LH. Data curation, writing original draft, and writing–review and editing: M-LH and DJ. Formal analysis: DJ. All authors have read and agreed to the published version of the manuscript.

## Conflict of Interest

The authors declare that the research was conducted in the absence of any commercial or financial relationships that could be construed as a potential conflict of interest.

## Publisher's Note

All claims expressed in this article are solely those of the authors and do not necessarily represent those of their affiliated organizations, or those of the publisher, the editors and the reviewers. Any product that may be evaluated in this article, or claim that may be made by its manufacturer, is not guaranteed or endorsed by the publisher.
